# Dynamic functional connectivity changes in the triple networks and its association with cognitive impairment in hemodialysis patients

**DOI:** 10.1002/brb3.2314

**Published:** 2021-08-01

**Authors:** Jianghui Cao, Guangzhi Liu, Xuekun Li, Zheng Yue, Jipeng Ren, Wei Zhu, Baolin Wu

**Affiliations:** ^1^ Department of Radiology, Xiangyang No. 1 People's Hospital Hubei University of Medicine Xiangyang China; ^2^ Department of Neurology Xiangyang No. 1 People's Hospital Hubei University of Medicine Xiangyang China; ^3^ Department of Magnetic Resonance The First Affiliated Hospital of Xinxiang Medical University Weihui China

**Keywords:** central executive network, default mode network, dynamic functional connectivity, end‐stage renal disease, hemodialysis, salience network

## Abstract

**Introduction:**

Cognitive impairment is common in hemodialysis (HD) patients; however, the underlying mechanisms have not been fully understood. The "triple‐network model" that consists of the salience network (SN), central executive network (CEN), and default mode network (DMN) has been suggested to play an important role in various cognitive functions. However, dynamic functional connectivity (FC) alterations within the triple networks have not been investigated in HD patients.

**Methods:**

Sixty‐six HD patients and 66 healthy controls (HCs) were included in this study. The triple networks were identified using a group spatial independent component analysis, and dynamic FC was analyzed using a sliding window approach and *k*‐means clustering algorithm. Furthermore, we analyzed the relationships between altered dynamic FC parameters and clinical variables in HD patients.

**Results:**

The intrinsic brain FC within the triple networks was clustered into four configuration states. Compared with HCs, HD patients spent more time in State 1, which was characterized by weak connections between the DMN and CEN and SN. HD patients showed lower number of transitions across different states than HCs. Moreover, the number of transitions and mean dwell time in State 1 were associated with cognitive performance in HD patients.

**Conclusion:**

Our findings suggest abnormal dynamic FC properties within the triple networks in HD patients, which may provide new insights into the pathophysiological mechanisms of their cognitive deficits from the perspective of dynamic FC.

## Graphical abstract

Cognitive impairment is common in hemodialysis (HD) end‐stage renal disease patients; however, the underlying mechanisms have not been fully understood. Here, we investigated dynamic functional connectivity (FC) in HD patients based on the "triple‐network model." We found that HD patients had altered dynamic FC properties, which were associated with their cognitive deficits.

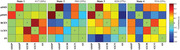

## 1 INTRODUCTION

Chronic kidney disease (CKD) is a substantial health problem worldwide, affecting 10−12% of the population (Levin et al., 2017). End‐stage renal disease (ESRD) is the terminal stage of CKD, and usually requires maintenance hemodialysis (HD) to sustain life over the long term (Webster et al., 2017). Notably, cognitive impairment (CI) is extremely common in HD patients, across numerous domains, particularly in the domains of orientation and attention and executive function, and patients often experience multiple deficits simultaneously (Drew et al., 2017; O'Lone et al., 2016; van Zwieten et al., 2018). Cognitive deficits are associated with poorer quality of life, risk for hospitalization, and increased mortality in HD patients (Drew et al., 2015; O'Lone et al., 2016; van Zwieten et al., 2019). However, up to now, the underlying pathophysiological mechanisms of CI in HD patients have not been completely clarified.

Resting‐state functional magnetic resonance imaging (Rs‐fMRI)‐based functional connectivity (FC) analysis, which quantifies intrinsic functional brain organization (Biswal et al., 1995), has become a valuable and non‐invasive tool to investigate the network connectivity basis of cognitive deficits in HD patients. For example, widespread weakening of cortical and subcortical network connectivity has been identified in HD patients (G. Zheng et al., 2014). The highly influential "triple‐network model," which was proposed by Menon (2011), provides a common framework for understanding the dysfunction in core neurocognitive networks across multiple psychiatric and neurological disorders. This model integrates three key intrinsic brain networks—the default mode network (DMN), central executive network (CEN), and the salience network (SN) into a single cohesive model that serves as the basis for normal behavior and cognition. Evidences from previous studies have demonstrated impaired interactions of the SN‐centered "triple‐network model" in various brain diseases, such as Alzheimer's disease (C. Li et al., 2019) and major depression disorder (H. Zheng et al., 2015). Our recent study of large‐scale network analysis using graph theory‐based approaches have demonstrated disrupted topological organizations of brain functional networks in patients with ESRD (Yue et al., 2021); however, we did not focus on cross‐network interactions of the triple networks. More importantly, this previous whole‐brain FC study on ESRD patients assumed that the FC was static within the entire RS‐fMRI scan, and did not consider the important dynamic aspect over time.

In recent years, more and more studies have focused on the dynamic changes of intrinsic FC across large‐scale brain functional networks. Dynamic functional network connectivity has been identified and explored in neuroimaging studies of healthy subjects, which was associated with higher‐order cognitions (Kucyi et al., 2017; Shine et al., 2019; Soreq et al., 2019). Moreover, abnormal dynamic brain functional network connectivity has also been demonstrated in clinical patients, such as those with bipolar disorder (Wang et al., 2019), Parkinson's disease (Kim et al., 2017), and autism spectrum disorder (Rashid et al., 2018). In addition, researchers have revealed abnormal dynamic FC in the triple networks in bipolar and major depressive disorders (Wang et al., 2020), providing new biomarkers for understanding their neural physiopathology. However, up to date, no studies have investigated the alterations of dynamic functional network connectivity in ESRD patients undergoing HD.

Considering the important roles of the triple networks in cognitive, perceptual, affective, and social functions and the important dynamic aspect of FC over time, we hypothesized that HD patients had abnormal dynamic FC in the triple networks and the altered dynamic FC properties might be the neural mechanisms underlying their cognitive dysfunction. To test our hypotheses, we investigated the dynamic FC alterations in HD patients compared with healthy controls (HCs) using a spatial group independent component analysis (ICA), a sliding window approach and *k*‐means clustering algorithm, and explored the underlying neural mechanisms underlying CI in HD patients.

## 2 METHODS

### 2.1 Participants

This prospective study was approved by the local Ethics Committee and followed the ethical guidelines of the Declaration of Helsinki; written informed consent was acquired from each subject before inclusion. A subset of our prior study sample (Yue et al., 2021) was included in this new analysis. In total, 66 patients with ESRD who received maintenance HD thrice weekly (HD group; 32 males and 34 females; mean age 33.03 ± 8.29 years, range from 19 to 45 years) were included in our study. Patients were included if they (1) had a history of chronic glomerulonephritis (disease duration > 6 months), and had reached ESRD with an estimated glomerular filtration rate of less than 15 mL/min per 1.73 m^2^; (2) received HD for at least 6 months; (3) aged > 18 years; and (4) right handed and had normal sight. The following exclusion criteria were used in our study: (1) history of drug abuse or alcohol addiction; (2) presence of organic brain lesions, such as tumor or stroke; (3) history of head trauma; (4) history of neurological or psychiatric disorders; (5) history of diabetic nephropathy, or hypertensive nephropathy, or nephrotic encephalopathy; (6) metabolic or cardiovascular diseases, such as diabetes and hypertension; and (7) head movement greater than 1.0 mm or 1.0°, or mean framewise displacement value exceeding the mean 0.2 mm.

Additionally, 66 age‐, sex‐ and education‐matched HCs (HC group; 36 males and 30 females; mean age 32.89 ± 7.82 years, range from 19 to 46 years) from the local community participated in the study. All HCs were right handed and had normal sight. The exclusion criteria used for the HC group were the same as those used for the HD group, and no HCs were excluded according to this exclusion criteria.

### 2.2 Neuropsychological tests

Before MR scanning, a battery of neuropsychological tests, including the Montreal Cognitive Assessment (MoCA), Trail Making Test A (TMT‐A), Symbol Digit Modalities Test (SDMT), and 17‐item version of the Hamilton Depression Rating Scale (HAMD) were performed by a neurologist (a non‐author with 15 years of experience in neuropsychological scale test). Specifically, the MoCA is a psychometrically valid instrument in assessment of the global cognitive function for all subjects. The TMT‐A is regarded as a useful valid measure of concentration, mental tracking, and audio visuomotor speed, and the SDMT is widely applied to measure psychomotor speed. Moreover, the HAMD is a primary and sensitive tool for the measurement of subjective depression level of individuals. Neuropsychological testing was performed the day after the patient's second dialysis session of the week. This was to avoid the immediate changes in cognition during and immediately after a dialysis session (Murray et al., 2007).

### 2.3 Laboratory examinations

Blood and urine tests were performed for all HD patients within 3 days before MRI examinations. The hemoglobin, serum creatinine, and blood urea nitrogen levels were recorded. No biochemistry tests were performed in HCs.

### 2.4 MRI data acquisition

MR imaging data were acquired using a 3T GE Discovery MR750 scanner (General Electric Healthcare, Milwaukee, WI) with a 16‐channel head coil. All subjects were instructed to be quiet, keep their eyes closed but stay awake, and try not to think about anything. First, conventional T_1_‐weighted, T_2_‐weighted, and T_2_‐weighted fluid‐attenuated inversion recovery imaging sequences were acquired to exclude organic brain lesions. Then, the Rs‐fMRI data were obtained using an echo‐planar imaging (EPI) sequence with the following parameters: 32 axial slices; repetition time (TR)/echo time (TE) = 2000/41 ms; field of view (FOV) = 240 × 240 mm^2^; matrix size = 64 × 64; slice thickness = 5 mm; slice gap = 0.4 mm; flip angle = 90°. Each functional image comprised 180 brain volumes and lasted 360 s. High‐resolution three‐dimensional T_1_‐weighted anatomical images were acquired using a three‐dimension brain volume imaging sequence with the following parameters: 188 sagittal slices; inversion time = 450 ms; TR/TE = 8.2/3.2 ms, FOV = 256 × 256 mm^2^; matrix size = 256 × 256; slice thickness = 1 mm; flip angle = 12°.

### 2.5 Image preprocessing

The Data Processing & Analysis for Brain Imaging toolbox (DPABI, version 4.1; http://rfmri.org/dpabi) (Yan et al., 2016) was used for pipeline data analysis of Rs‐fMRI. Briefly, the first 10 volumes of the Rs‐fMRI dataset of each subject were discarded to allow for MR signal equilibrium; thus, the remaining 170 volumes were used for further analyses. Then, the remaining Rs‐fMRI data were corrected for the temporal differences between slices, and were realigned to the first volume for the correction of head motion. Next, the corrected Rs‐fMRI data were spatial normalized to the standard Montreal Neurological Institute (MNI152) space (resampling voxel size = 3 × 3 × 3 mm^3^) by using diffeomorphic anatomical registration through exponentiated Lie algebra (DARTEL) (Ashburner, 2007). Finally, the normalized Rs‐fMRI images were spatially smoothed with a 6‐mm full‐width at half‐maximum Gaussian kernel.

### 2.6 Group ICA and identification of intrinsic brain networks

To decompose the preprocessed Rs‐fMRI data into different independent components (ICs), spatial group ICA was performed using the Group ICA of fMRI Toolbox (GIFT, version 4.0b; http://icatb.sourceforge.net). The ICA included three steps: (1) data reduction; (2) ICs decomposition; and (3) back reconstruction. Specifically, we first used a two‐step principal component analysis (PCA) to reduce the data into 37 ICs. This averaged IC number was determined using the minimum description length (MDL) criteria (Y. O. Li et al., 2007) and was used for each subject for ICA decomposition. Second, ICs estimation was performed using the Infomax algorithm (Bell & Sejnowski, 1995). In this step, the spatial map and the time course of blood oxygenation level‐dependent (BOLD) signal were generated for each IC. This step was repeated 100 times using the ICASSO algorithm to identify the most stable and reliable components (Himberg et al., 2004). Finally, subject‐specific spatial maps and time courses for each IC were obtained using group ICA back reconstruction algorithm (Calhoun et al., 2001), and the subject‐specific maps were converted to *z*‐scores.

Subsequently, all ICs were evaluated based on the group IC maps by following the criteria suggested by previous studies (Allen et al., 2014; Cordes et al., 2000; Kim et al., 2017): (1) peak activations of spatial maps located in gray matter; (2) low spatial overlap with known vascular, ventricular, motion, and susceptibility artifacts; (3) time courses dominated by low‐frequency signals (ratio of powers below 0.1 Hz to 0.15−0.25 Hz in spectrum); and (4) time courses characterized by a high dynamic range (a range difference between the minimum and maximum power frequencies). Using the spatial sorting function of GIFT, we selected five ICs that correspond to the anterior DMN (aDMN), posterior DMN (pDMN), left CEN (LCEN), right CEN (RCEN), and SN, based on the maximum spatial correlation with the spatial network template (Shirer et al., 2012).

To remove physiological and scanner noise sources, we performed post‐processing steps to the time courses of ICs, which included: (1) detrending linear, quadratic, and cubic trends; (2) multiple regression of the six realignment parameters and their temporal derivatives; (3) removal of detected outliers; and (4) low‐pass filtering with a high‐frequency cutoff of 0.15 Hz. The outliers were detected based on the median absolute deviation, which is implemented in 3D DESPIKE. The outliers were replaced with the best estimate using a third‐order spline fit to the clean portion of the time courses. At last, the remaining time courses were used for further dynamic FC analyses.

### 2.7 Dynamic FC computation

A sliding window approach was applied for the computation of dynamic FC using the Temporal dFNC toolbox in GIFT. In each window, the Pearson's correlation coefficients of time courses of each pair of the five resting‐state networks were calculated, resulting in a 5 × 5 correlation matrix. We used a tapered window, created by convolving a rectangle (width = 30 TRs, i.e., 60 s) with a Gaussian (*σ* = 3 TRs), and the window was shifted with step size of 1 TR each time, resulting in 140 consecutive windows across the entire Rs‐fMRI scan. The window length of 30 TRs was chosen based on a previous study (Yu et al., 2015), which suggests that this window size may provide a good trade‐off between the quality of the FC estimate and the temporal resolution. To characterize the full covariance matrix, we estimated covariance from the regularized precision matrix or the inverse covariance matrix (Smith et al., 2011). To promote sparsity in estimation, a penalty on the L1 norm (i.e., the sum of the absolute values of the elements of the precision matrix) was imposed in the graphic LASSO framework with 10 repetitions (Friedman et al., 2008). Values in the resulting FC matrices were converted to *z*‐scores using Fisher's *r*‐to‐*z* transformation to improve the normality of the distribution of Pearson's *r* and were then residualized with known confounding variables, such as age and gender. The resulting individuals’ 140 FC matrices represent the dynamic changes of FC during the whole Rs‐fMRI scan period and were used for further FC state analysis.

### 2.8 Dynamic FC state analysis

#### 2.8.1 Clustering analysis

We applied a *k*‐means clustering algorithm on all the windowed 5 × 5 FC matrices for all subjects to estimate reoccurring FC patterns (states) with a random initialization of the centroid positions. The *k*‐means clustering algorithm was repeated 500 times to increase the chance of escaping the local minima. The optimal number of clusters was estimated to be four (*k* = 4) using the elbow criterion (Allen et al., 2014). The correlation distance method was chosen because it is more sensitive to the dynamic FC pattern irrespective of magnitude (Rashid et al., 2018). Subsequently, these resulting centroids were used as starting points to cluster the dynamic FC windows for all the subjects. From these data, we obtained a state transition vector representing their state status across time. Final cluster centroids were obtained as the median of all state‐assigned FC matrices across time.

#### 2.8.2 Temporal properties

We calculated three temporal properties of dynamic FC states derived from each subject's state vector, including the reoccurrence fraction and mean dwell time in each state, as well as the total number of transitions across different states. The interpretation and meaning of these three dynamic FC properties were as follows (Kim et al., 2017): (1) fractional window is the proportion of time spent in each state as measured by percentage; (2) the mean dwell time represents how long the participant stayed in a certain state, which was calculated by averaging the number of consecutive windows belonging to one state before changing to the other state; and (3) the number of transitions represents how many times either state changed from one to the other, counting the number of times a switch occurred, with more transitions representing less stability over time.

#### 2.8.3 FC strength

The subject‐specific centroid of each state was computed by calculating the median value of each FC matrix for that state. To determine between‐group differences in connectivity strength, we calculated the group‐specific centroids of the four states by averaging subject‐specific centroids of both HD patients and HCs, respectively.

### 2.9 Statistical analysis

#### 2.9.1 Group differences in demographic and clinical data

A chi‐square test was used to determine gender‐ratio difference between the HD group and HC group, and an independent two‐sample *t*‐test was used to compare the clinical variables and neuropsychological test results between the two groups. Statistical analysis was performed using software (SPSS, version 21.0; IBM Corp, Armonk, NY). Statistical significance was defined as *p *< .05.

#### 2.9.2 Group differences in dynamic FC

Between‐group differences in temporal properties of dynamic FC states strength were tested using a Mann–Whitney *U*‐test in SPSS software, and between‐group differences in FC strength of each state were tested using an independent two‐sample *t*‐test in GRETNA software (http://www.nitrc.org/projects/gretna). The significance level was set to false‐discovery rate‐corrected *p *< .05.

#### 2.9.3 Relationship between altered dynamic FC properties and clinical variables

A partial correlation analysis was used to estimate the relationships between those dynamic FC parameters showing significant between‐group differences and the clinical variables in the HD group (*p* < .05, uncorrected), with age, sex and educational level as covariables. The clinical variables comprised the neuropsychological test results, duration of HD, hemoglobin level, serum creatinine level, and serum urea level.

## 3 RESULTS

### 3.1 Demographic and clinical characteristics

The demographic and clinical characteristics are summarized in Table 1. There were no significant differences in age (*p* = .923), sex (*p* = .486), and education level (*p* = .295) between the two groups. The MoCA (*p *< .001) and SDMT (*p* < .001) scores of the HD group were significantly lower than those of the HC group, and HD patients spent longer time in the completion of TMT‐A than HCs (*p* < .001). Moreover, the HAMD‐17 score of the HD group was significantly higher than that of the HC group (*p* < .001).

**TABLE 1:** Demographics and clinical characteristics of HD patients and healthy controls

	HD (*n *= 66)	HCs (*n *= 66)	*p* value
Sex (M/F)	32/34	36/30	.486^†^
Age (year)	33.03 ± 8.29	32.89 ± 7.82	.923^‡^
Education (year)	11.26 ± 3.09	11.83 ± 3.19	.295^‡^
Disease duration (month)	28.83 ± 6.36	—	—
Dialysis duration (month)	15.76 ± 6.11	—	—
Hemoglobin (g/L)	91.25 ± 23.26	—	—
Serum creatinine (μmol/L)	732.43 ± 203.09	—	—
Urea (mmol/L)	20.04 ± 8.15	—	—
MoCA (score)	22.47 ± 4.07	27.45 ± 1.11	<.001^‡^
TMT‐A (s)	70.58 ± 18.17	55.67 ± 19.68	<.001^‡^
SDMT (score)	38.77 ± 9.90	47.97 ± 10.62	<.001^‡^
HAMD‐17 (score)	17.27 ± 4.00	12.93 ± 1.96	<.001^‡^

*Note*.—All quantitative data are expressed as mean ± standard deviation; numbers for sex data.

^†^ The *p* value was calculated by using chi‐square test.

^‡^ The *p* value was calculated by using independent two‐samples *t*‐test.

Abbreviations: HAMD‐17, 17‐item version of the Hamilton Depression Rating Scale.; HCs, healthy controls; HD, hemodialysis; MoCA, Montreal Cognitive Assessment; SDMT, Symbol Digit Modalities Test; TMT‐A, Trail Making Test A.

### 3.2 Intrinsic FC networks

**FIGURE 1:**
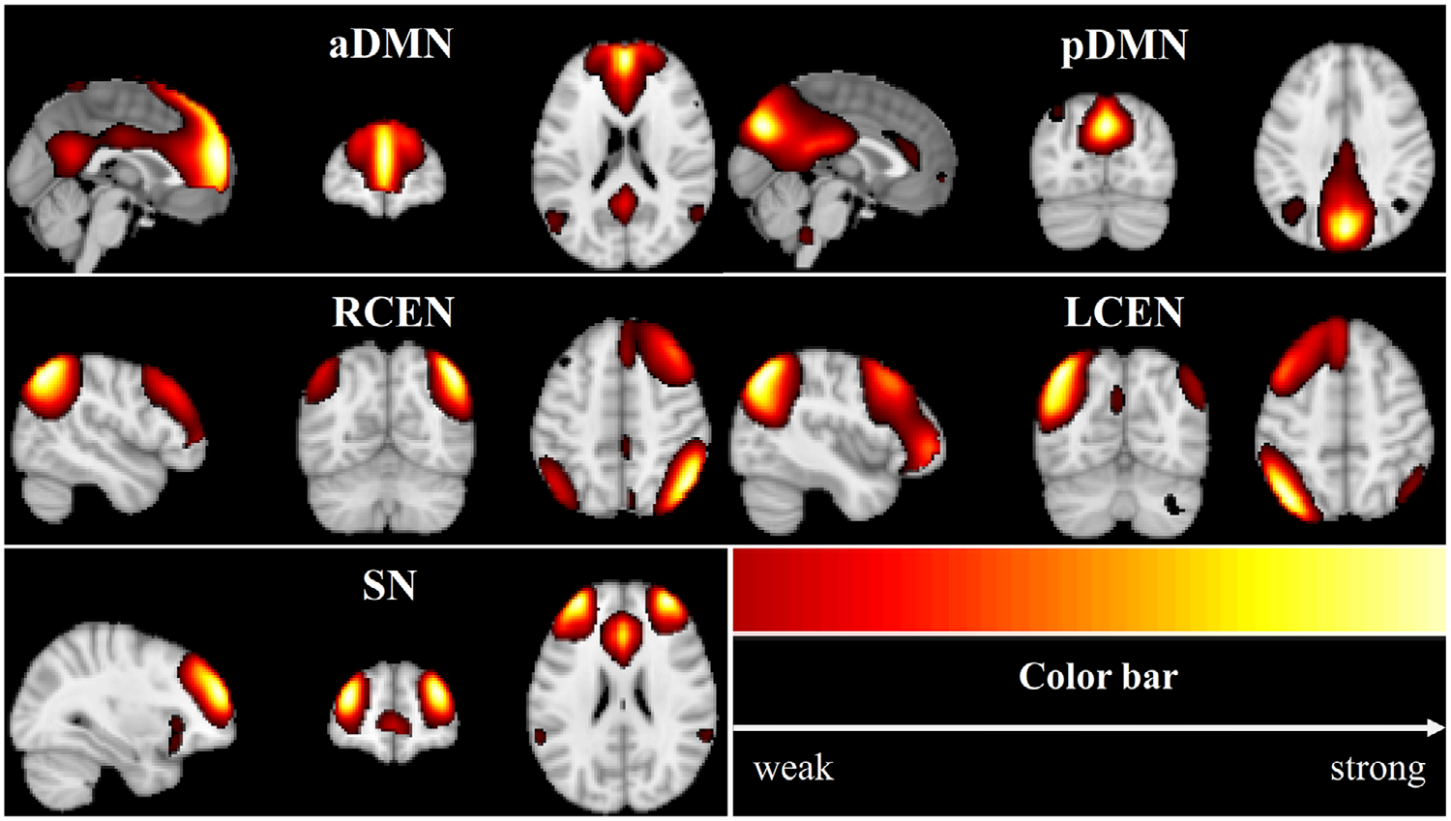
Spatial distribution maps for the five identified independent components, which correspond to the anterior default mode network (aDMN), posterior default mode network (pDMN), right central executive network (RCEN), left central executive network (LCEN), and salience network (SN)

As shown in Figure 1, the five ICs, aDMN (IC12), pDMN (IC8), RCEN (IC27), LCEN (IC20), and SN (IC8), were selected from the 37 ICs. The spatial maps of those ICs were similar to those found in previous studies (Liu et al., 2021; Wang et al., 2020) and covered most of the grey matter.

### 3.3 Dynamic FC state analysis

**FIGURE 2:**
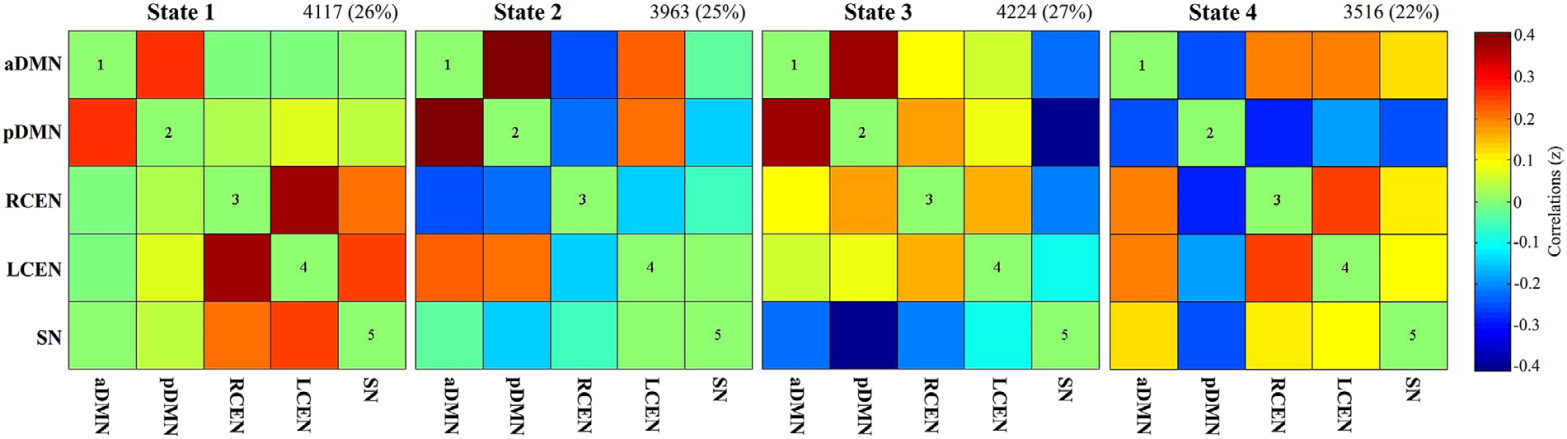
Cluster centroid and its total number of occurrence (percentage) for each state. The color bar represents *z* value of functional connectivity. Abbreviations: aDMN, anterior default mode network; LCEN, left central executive network; pDMN, posterior default mode network; RCEN, right central executive network; SN, salience network

Using the *k*‐means clustering algorithm, we identified four highly structured FC states that recurred throughout individual scans and across subjects (Figure 2). In State 1, which accounts for 26% of all windows, the DMN showed negative FC with CEN and SN, and the SN had positive FC with CEN. In State 2, which accounts for 25% of all windows, the DMN had negative FC with RCEN and SN but positive FC with LCEN. In State 3, which accounts for 27% of all windows, the DMN had positive FC with CEN but negative FC with SN, and the SN had negative FC with CEN. In State 4, which only accounts for 22% of all windows, the CEN had positive FC with aDMN and SN but negative FC with pDMN, and the SN had positive FC with aDMN but negative FC with pDMN. Notably, the FC values between the DMN and CEN and SN in State 1 are much weaker than in other states.

**FIGURE 3:**
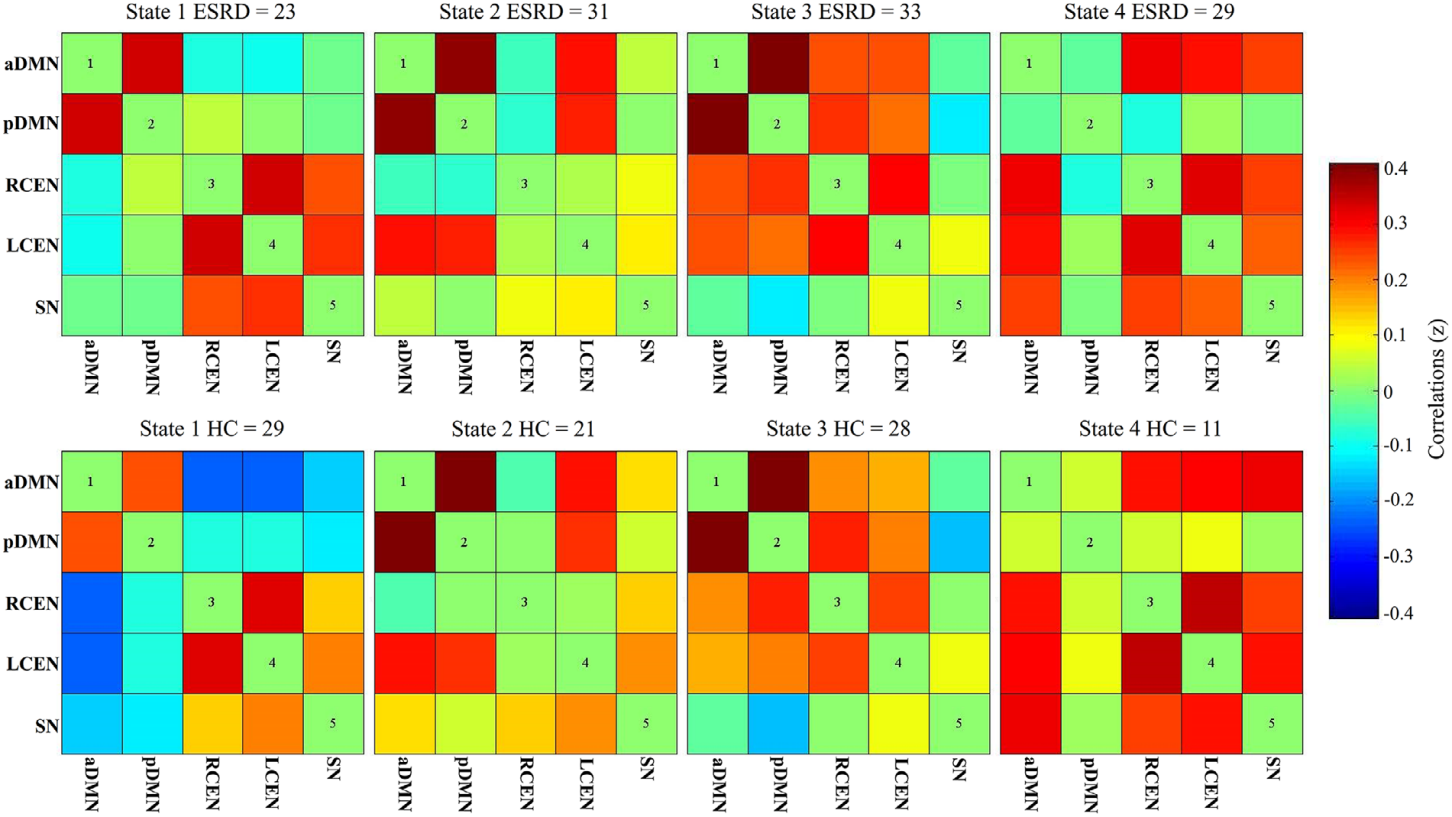
Group‐specific centroid matrices for each state. The color bar represents *z* value of functional connectivity. Abbreviations: aDMN, anterior default mode network; LCEN, left central executive network; pDMN, posterior default mode network; RCEN, right central executive network; SN, salience network

The group‐specific medians for each state are shown in Figure 3. Notably, not all subjects had the windows assigned to each state, which contributed to changes of the number of subject‐specific matrices in different states (see Figure 3 for subject counts of each state). No significant between‐group differences in FC strength were found in each state after FDR correction.

**FIGURE 4:**
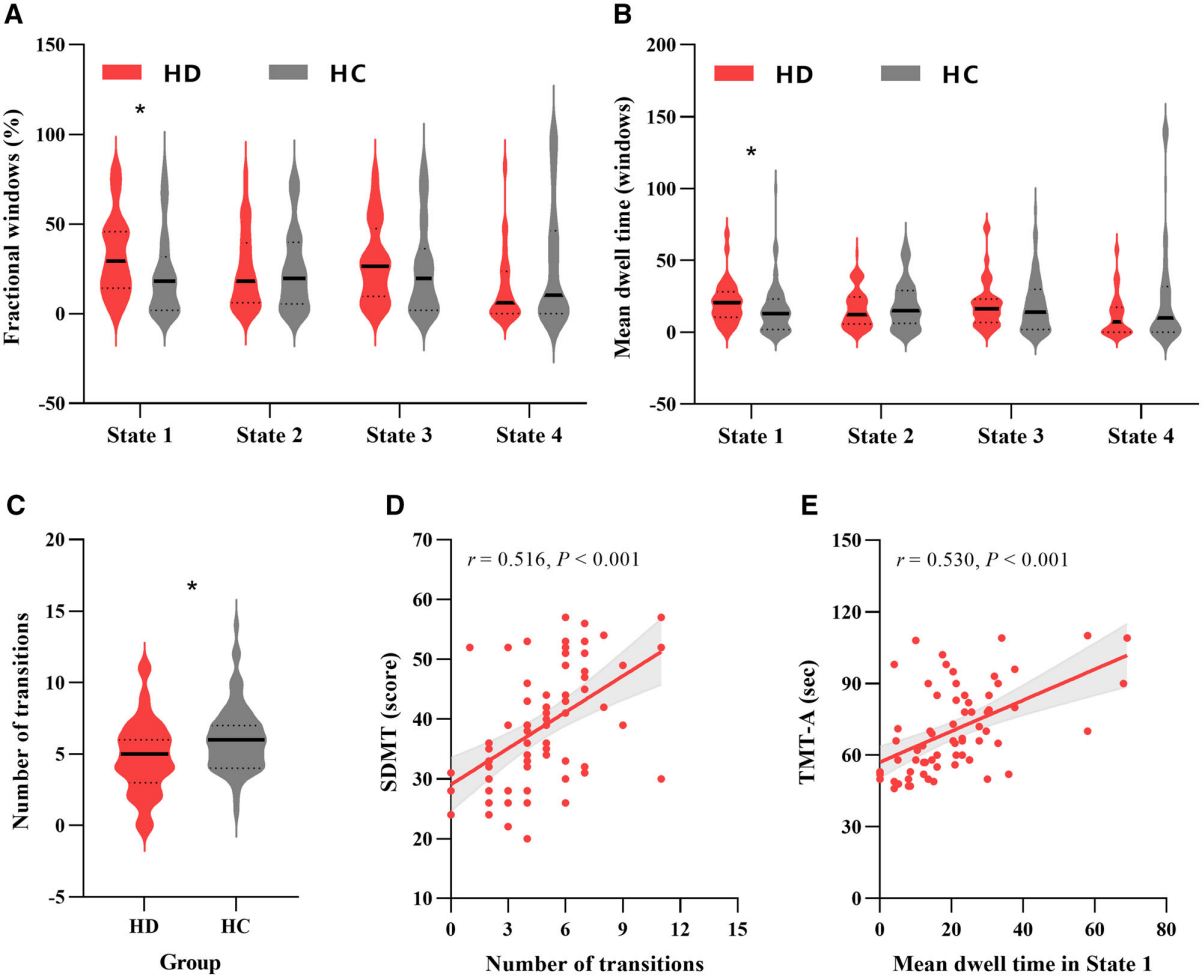
Between‐group comparison of temporal properties and the partial correlation analysis results. The temporal properties (a–c) of functional connectivity state analysis are shown using violin plots. Transverse solid and dotted lines represent medians and quartiles, respectively. Asterisks indicate a significant between‐group difference (*p* < .05, FDR corrected). For hemodialysis patients, (d) the number of transitions positively correlated with the SDMT score , (e) and the mean dwell time in State 1 positively correlated with the completion time of TMT‐A. Abbreviations: SDMT, Symbol Digit Modalities Test; TMT‐A, Trail Making Test A

Between‐group differences in temporal properties of FC states are shown in Table 2 and Figure 4a–c. HD patients had significantly higher reoccurrence fraction (*p* = .009, FDR corrected) and longer mean dwell time (*p* = .039, FDR corrected) in State 1 compared with HCs. Moreover, the HD group showed significantly lower number of transitions across states compared with the HC group (*p* = .014, FDR corrected).

**TABLE 2:** Between‐group differences in temporal properties of dynamic functional connectivity states

	HD Patients		Healthy controls		Statistics	
	Median	Interquartile range	Median	Interquartile range	*z* value	*p* value*
Fractional windows (%)						
State 1	29.29	(14.29, 45.71)	18.21	(1.96, 31.79)	−3.201	.009
State 2	18.21	(6.07, 39.46)	19.64	(5.54, 39.82)	−0.009	.993
State 3	26.43	(9.64, 47.50)	19.64	(1.96, 36.25)	−1.445	.234
State 4	6.07	(0.00, 23.57)	10.36	(0.00, 46.25)	−1.736	.185
Mean dwell time (windows)						
State 1	20.50	(10.37, 28.23)	13.00	(1.88, 23.13)	−2.488	.039
State 2	12.25	(5.75, 24.69)	15.13	(6.25, 29.08)	−0.620	.603
State 3	16.33	(6.75, 23.17)	14.00	(2.00, 30.00)	−0.782	.558
State 4	7.25	(0, 17.50)	10.00	(0.00, 31.88)	−1.418	.234
Number of transitions (time)	5.00	(3.00, 6.00)	6.00	(4.00, 7.00)	−2.957	.014

* Statistical results were corrected with false‐discovery rate.

Abbreviation: HD, hemodialysis.

### 3.4 Associations between altered dynamic FC properties and clinical variables

For the HD group, the number of transitions across states positively correlated with the SDMT score (*r* = 0.516, *p* < .001, uncorrected) (Figure 4d), and the mean dwell time in State 1 positively correlated with the completion time of TMT‐A (*r* = 0.530, *p* < .001, uncorrected) (Figure 4e). No significant correlations were found between altered dynamic FC properties and other clinical variables.

## 4 DISCUSSION

By using Rs‐fMRI in combination with clustering algorithm and FC state analysis, the present work is the first study to analyze the dynamic FC alterations in HD patients based on the "triple‐network model," focusing on the temporal properties and FC strength of dynamic FC states. Three main findings were found in our study. First, the dynamic FC within the triple networks could be clustered into four discrete connectivity configurations across all subjects. In particular, State 1 was characterized by weak connections between the DMN and SN and CEN. Second, the dynamic FC state analysis revealed that HD patients spent longer time in State 1 and switched less frequently across states compared with HCs. Third, the altered dynamic FC properties were associated with cognitive performance in HD patients. These findings provide new insights into the pathophysiological mechanisms underlying CI in HD patients, and underscore the importance of evaluating dynamic changes of brain connectivity.

An important finding of our study was the decreased ability to switch between different FC states within the triple networks in HD patients, as characterized by the lower number of state transitions. HCs dynamically switch between different FC states and are therefore probably faster in recruiting necessary resources in the face of changing task demands (Yu et al., 2015). Thus, a lower total number of transitions across states in HD patients may suggest a slower speed to recruit necessary resources when faced with different cognitive tasks, which may lead to the decline in cognitive performance. Indeed, recent studies have demonstrated the association between network flexibility and cognitive task performance (Garrett et al., 2013; Madhyastha et al., 2015; Spreng & Schacter, 2012; Thompson et al., 2013). Furthermore, the positive correlation between number of transitions and SDMT scores revealed that worsening of cognitive function was associated with decreasing of the total number of transitions in those patients. Therefore, this study may suggest that the dynamic FC properties, especially the number of transitions, may act as a reliable marker for monitoring the progression of CI in HD patients.

Another important finding of this study was that HD patients, relative to controls, spent more time in State 1. Notably, the FC between the DMN and CEN and SN are weaker than in other states. It is reported that the interactions within the triple networks are responsible for maintaining information processing, such as cognitive, perceptual, affective, and social functions (Menon, 2011). Thus, HD patients who spent longer time in State 1 might reflect a decreased ability of information communication among different networks. In fact, our further correlation analysis revealed that the mean dwell time in State 1 was positively associated with the completion time of TMT‐A, which is widely used to measure executive function, concentration, mental tracking, and audio visuomotor speed. Considering the existed decreased information communication ability across states, our results may suggest that the decreased information communication is state independent in HD patients.

We acknowledge several limitations of this study. First, the dynamic FC analysis is a relatively new approach, and the gold standard in the setting of analysis parameters, such as window length and overlap, has not been established. This inconsistency in setting parameters may affect the analysis results. Second, the Rs‐fMRI scanning parameters should be mentioned when conducting dynamic FC analysis. It is considered that high temporal resolution and a sufficient length of acquisition are both important factors for reliable results in the dynamic FC analysis (Kim et al., 2017). In the present study, we used a typical TR (TR = 2s) to acquire Rs‐fMRI images. Previous studies have demonstrated the reliability of this typical TR in sampling the dynamics of low‐frequency fluctuation (Allen et al., 2018; Liao et al., 2014). However, to increase the estimation power of FC matrices calculated within small windows in the sliding window approach, it would be beneficial to use a rapid Rs‐fMRI acquisition, such as the simultaneous multi‐band multi‐slice EPI acquisition (Moeller et al., 2010), that could sample more dense time series. Third, although we found an association between the number of transitions and the SDMT score, one might have expected the number of transitions to be associated also with the completion time of TMT‐A given its relevance to processing speed. Actually, our partial correlation analysis revealed a negative correlation between these two measures; however, this result was not significant, which might be attributed to the relatively small sample size. Finally, the sample size of our study was relatively small, which may affect the statistical power. Future studies with large sample sizes are needed to verify the reproductivity of our findings.

## 5 CONCLUSIONS

In summary, the present study investigated the dynamic FC properties in the triple networks in HD patients using Rs‐fMRI data in combination of ICA, sliding windows approach, and *k*‐means clustering analysis. Our study demonstrated altered dynamic FC properties in the triple networks in HD patients compared with HCs, including increased time in the weakly connected State 1 and decreased number of transitions. In addition, the altered dynamic FC properties were associated with cognitive performance in HD patients. Thus, this study provides new insights into the pathophysiological mechanisms of CI in HD patients from the perspective of dynamic FC.

## CONFLICT OF INTEREST

The authors declare no conflict of interest.

## AUTHOR CONTRIBUTIONS

Baolin Wu conceived and designed the study, supervised the conduct of the study, and took responsibility for the paper. Xuekun Li, Zheng Yue, and Jipeng Ren were responsible for data acquisition. Jianghui Cao and Guangzhi Liu analyzed the data. Xuekun Li assisted with the literature review. Jianghui Cao and Guangzhi Liu drafted the initial manuscript. Baolin Wu and Wei Zhu reviewed and revised the manuscript. All authors read and approved the final manuscript.

### PEER REVIEW

The peer review history for this article is available at https://publons.com/publon/10.1002/brb3.2314

## Data Availability

The data that support the findings of this study are available from the corresponding author upon reasonable request.
